# Causes of acute undifferentiated fever and the utility of biomarkers in Chiangrai, northern Thailand

**DOI:** 10.1371/journal.pntd.0006477

**Published:** 2018-05-31

**Authors:** Tri Wangrangsimakul, Thomas Althaus, Mavuto Mukaka, Pacharee Kantipong, Vanaporn Wuthiekanun, Wirongrong Chierakul, Stuart D. Blacksell, Nicholas P. Day, Achara Laongnualpanich, Daniel H. Paris

**Affiliations:** 1 Mahidol-Oxford Tropical Medicine Research Unit (MORU), Faculty of Tropical Medicine, Mahidol University, Bangkok, Thailand; 2 Centre for Tropical Medicine and Global Health, Nuffield Department of Clinical Medicine, University of Oxford, Oxford, United Kingdom; 3 Department of Medicine, Chiangrai Prachanukroh Hospital, Chiangrai, Thailand; 4 Faculty of Tropical Medicine, Mahidol University, Bangkok, Thailand; 5 Department of Medicine, Swiss Tropical and Public Health Institute, Basel, Switzerland; 6 Faculty of Medicine, University of Basel, Basel, Switzerland; International Vaccine Institute, REPUBLIC OF KOREA

## Abstract

**Background:**

Tropical infectious diseases like dengue, scrub typhus, murine typhus, leptospirosis, and enteric fever continue to contribute substantially to the febrile disease burden throughout Southeast Asia while malaria is declining. Recently, there has been increasing focus on biomarkers (i.e. C-reactive protein (CRP) and procalcitonin) in delineating bacterial from viral infections.

**Methodology/Principal findings:**

A prospective observational study was performed to investigate the causes of acute undifferentiated fever (AUF) in adults admitted to Chiangrai Prachanukroh hospital, northern Thailand, which included an evaluation of CRP and procalcitonin as diagnostic tools. In total, 200 patients with AUF were recruited. Scrub typhus was the leading bacterial cause of AUF (45/200, 22.5%) followed by leptospirosis (15/200, 7.5%) and murine typhus (7/200, 3.5%), while dengue was the leading viral cause (23/200, 11.5%). Bloodstream infections contributed to 7/200 (3.5%) of the study cohort. There were 9 deaths during this study (4.5%): 3 cases of scrub typhus, 2 with septicaemia (*Talaromyces marneffei* and *Haemophilus influenzae*), and 4 of unknown aetiologies. Rickettsioses, leptospirosis and culture-attributed bacterial infections, received a combination of 3^rd^ generation cephalosporin plus a rickettsia-active drug in 53%, 73% and 67% of cases, respectively. Low CRP and white blood count were significant predictors of a viral infection (mainly dengue) while the presence of an eschar and elevated aspartate aminotransferase and alkaline phosphatase were important predictors of scrub typhus.

**Interpretation:**

Scrub typhus and dengue are the leading causes of AUF in Chiangrai, Thailand. Eschar, white blood count and CRP were beneficial in differentiating between bacterial and viral infections in this study. CRP outperformed procalcitonin although cut-offs for positivity require further assessment. The study provides evidence that accurate, pathogen-specific rapid diagnostic tests coupled with biomarker point-of-care tests such as CRP can inform the correct use of antibiotics and improve antimicrobial stewardship in this setting.

## Author summary

Fever remains an important reason why people are hospitalised in Southeast Asia. We do not know the most common causes of fever in many regions of the tropics. This knowledge would help doctors decide on the most appropriate treatment in areas where access to diagnostics is difficult. Establishing diagnostic tests for all possible diseases in an area is expensive and often impractical. An alternative is to measure ‘marker’ chemicals in the blood which the body produces in response to infection. These are usually higher in patients with bacterial infections. Differentiating bacterial from viral infections will help reduce inappropriate antibiotic use, which can contribute to the development of antibiotic-resistant bacteria. In this study, we investigated the causes of fever in hospitalised patients in Chiangrai, northern Thailand, and assessed if two chemical markers (CRP and procalcitonin) could distinguish bacterial from viral infections. Scrub typhus, dengue and leptospirosis were the major causes of fever, and these were not always accurately diagnosed and managed. We also found that CRP was better than procalcitonin in differentiating bacterial from viral infections. These results should help improve the management of febrile patients and increase the awareness of these neglected tropical diseases that are potentially deadly if missed.

## Introduction

Acute undifferentiated fever (AUF) remains the leading cause of hospitalisation among adults and children in urban and rural regions of Southeast Asia. The causes include common diseases such as dengue, scrub typhus, murine typhus, leptospirosis, and enteric fever, which continue to contribute significantly to the febrile disease burden [1–4]. Although malaria may present similarly, its overall incidence and impact on health in this region is declining [5].

In Laos, a prospective multicentre study investigating the causes of non-malarial fever revealed dengue, scrub typhus, Japanese encephalitis and leptospirosis as the major aetiologies in hospitalised adults and children once influenza was excluded [6]. In rural Thailand, dengue, scrub typhus, leptospirosis, murine typhus, and influenza have been identified as the most common causes of AUF among adults and children [4, 7]. Scrub typhus, enteric fever, flavivirus infection, leptospirosis and malaria were the main causes of fever in adults and children in the 1970s in rural Malaysia [8]. In febrile pregnant women on the Thai-Burmese border and in Laos, malaria, rickettsial infections, dengue, leptospirosis, typhoid and pyelonephritis predominate [9, 10]. Adverse neonatal and maternal outcomes were high in this group, particularly in those diagnosed with rickettsial infections [10, 11]. In Cambodian children, dengue, scrub typhus, bacteraemia (*Salmonella enterica* serovar Typhi was the commonest pathogen) and Japanese encephalitis were the major diagnoses [3].

These “causes-of-fever” studies that address a wide range of infectious diseases in diverse geographies are useful in informing clinicians and epidemiologists alike [12]. However, the majority of currently available fever studies suffer from selection bias, often rely on suboptimal diagnostic tools and non-uniform positivity criteria—limiting estimates of disease incidence and burden [13]. Performing these prospective studies correctly is costly, difficult and challenging–especially if representative geographical coverage is desired [12]. The current literature highlights a panel of AUF that represents the leading causes of fever in Asia and similarities in their clinical presentation and poor access to high-quality, affordable diagnostic tools frequently result in sub-optimal management [14]. Although progress in developing accurate, validated and cost-effective diagnostic tools for non-malarial pathogens, such as disease-specific rapid diagnostic tests (RDTs), have been made (e.g. combining NS1-antigen with IgM detection in dengue), recent modelling approaches suggest that testing for viral infections is unlikely to be cost-effective when considering direct health benefits, whereas RDTs for the detection of prevalent bacterial pathogens could be [15, 16].

Biomarkers such as C-reactive protein (CRP) and procalcitonin have some utility in delineating between bacterial and viral infections and guiding healthcare workers on the appropriate use of antibiotics in patients with respiratory tract infections in high income settings [17]. A retrospective study based on well-characterised samples of adults and children with febrile illnesses from Cambodia, Laos and Thailand demonstrated CRP was highly sensitive and moderately specific for discriminating between bacterial and viral infections [18]. Recently, CRP testing has been shown to reduce antibiotic prescription for acute respiratory illnesses in adults and children in primary healthcare settings in Vietnam [19]. In resource-constrained tropical settings, common treatable infections are being missed and inappropriate use of antibiotics is widespread. This highlights the potential impact of CRP RDTs on the precision of antibiotic use and contribution to the global strategy to combat antimicrobial resistance.

In this prospective study, we investigated the causes of AUF in adults admitted to the provincial hospital in Chiangrai, northern Thailand, and evaluated the use of CRP and procalcitonin tests in guiding appropriate antibiotic use.

## Materials and methods

### Ethics statement and study site

The Chiangrai Hospital Ethical Committee, Thai Ministry of Public Health and the Faculty of Tropical Medicine Ethics Committee, Mahidol University, Bangkok, granted ethical approval for this study (MUTM 2006–035). All patients provided written informed consent prior to sample collection, and parents or guardians provided informed consent on behalf of all child participants. Chiangrai Prachanukroh hospital is located in Chiangrai province, the northernmost province in Thailand, and near “the Golden Triangle” where Thailand, Laos and Myanmar converge. The province population of 1.2 million consists mainly of ethnic Thais with 12.5% belonging to hill tribes and other minority ethnic groups.

### Patient data and samples

Between August 2006 and October 2008, we prospectively recruited a total of n = 231 patients age ≥15 years old at Chiangrai Prachanukroh hospital with a fever >37.5°C or a history of fever within the past 21 days, no evidence of a primary focus of infection (e.g. consolidation on chest X-ray, symptoms and signs of a urinary tract infection, cellulitis) and negative for malaria on blood film. Demographic, clinical and laboratory data related to the admission were collected individually on study case-record forms (CRFs) from patient notes and hospital records. Demographic data included age, sex, and occupation. A rural/agricultural occupation was defined as those working as farmers, gardeners, agricultural/plantation workers, or fish and animal farm workers. Clinical data included symptoms, examination findings and vital signs on admission along with details of the current illness, prior antibiotic use, antibiotic treatment during admission, and illness outcome (e.g. fever days, death). Laboratory data included haematology (complete blood count) and biochemistry (renal and liver blood tests) results from admission samples. Chest x-ray findings were also recorded if performed.

An acute study blood sample was collected by study staff on enrolment in addition to the routine tests requested by the treating physician (10ml EDTA whole blood and 10ml clotted blood for serum). Blood and other routine cultures were performed if requested by the local clinician and processed using conventional techniques at the hospital microbiology laboratory. HIV testing was performed as part of routine hospital work using RDTs at the discretion of the treating physician. Follow-up was carried out by study staff 7–14 days after enrolment and involved a clinical review and collection of a convalescent blood sample (10ml clotted blood for serum).

There were 19 patients with incomplete CRFs/datasets and 12 patients with incomplete sample collections. These 31 patients were excluded resulting in a total of 200 study eligible patients. Of these, 171/200 (86%) provided paired samples obtained on admission and follow-up between days 7–14, and 29/200 patients had a confirmatory diagnosis made from admission samples alone. Both admission and follow-up samples were used for the diagnostic assays outlined below. Inflammatory biomarkers were tested on acute samples only. The clotted blood samples were processed for serum, aliquoted, stored locally at -30°C, and batch transported on dry-ice for storage (-80°C) and subsequent analysis at the central laboratory of Mahidol-Oxford Tropical Medicine Research Unit (MORU) in Bangkok. EDTA whole blood samples were transported at ambient temperature on the day of collection to Bangkok for further analysis. Some whole blood samples were processed immediately for culture for leptospirosis and scrub typhus (see below) with the remainder stored as aliquots of whole blood, plasma and buffy coat at -80°C.

In addition, meteorological data comprising average monthly temperatures and total monthly rainfalls were retrospectively collected for the study period from the local Thai Meteorological Department office of Mueang district in Chiangrai province. The data was collected from the district’s weather station near the airport. Chiangrai Prachanukroh Hospital is located within this central district, which is its main catchment area, but the hospital also admits severely ill patients from surrounding districts.

### Diagnostic assays

The diagnostic panel included diagnosis of dengue, scrub typhus, murine typhus, leptospirosis and Japanese encephalitis. Dengue diagnosis was performed in paired sera using the following ELISA tests: PanBio Dengue Early NS1 (Alere), PanBio Dengue IgM capture (Alere), PanBio Dengue IgG capture (Alere), and PanBio Japanese Encephalitis/Dengue IgM combo (Alere). An admission titer ≥10 U of NS1 PanBio units and/or ≥4-fold increase of IgM antibodies in the convalescent sample was considered diagnostic of acute primary dengue virus infection. Patients with anti-JEV IgM levels of >40 U were classified as having acute JEV infections only if anti-dengue IgM levels were <40 U using the combination ELISA test. Leptospirosis culture was performed at MORU within 24–48 hours by injecting 100μL of whole blood and 200μL of plasma sediment (the bottom fraction obtained from centrifuging 500μL of heparinized plasma) into 3 mL of Ellinghausen, McCullough, Johnson, and Harris (EMJH) medium, supplemented with 3% rabbit serum and 0.1% agarose. Both culture tubes were incubated aerobically at 25°C–30°C and examined every week for 3 months for evidence of growth. The leptospirosis SD Bioline RDTs were used for detecting anti-leptospira IgM and IgG. Scrub typhus and murine typhus were diagnosed using the indirect immunofluorescence assay (IFA) to detect IgM antibody titers in paired sera (or in admission samples only if convalescent samples unavailable) against *Orientia tsutsugamushi* antigens (Karp, Kato and Gilliam strains for scrub typhus) and *Rickettsia typhi* antigens (Wilmington strain for murine typhus), respectively. The new diagnostic IFA cut-off titer of ≥1:3,200 in an admission sample or ≥4-fold rise to ≥1:3,200 in a convalescent-phase sample was used [20]. For scrub typhus, culture and polymerase chain reaction (PCR) assays were also performed as previously described [21]. Briefly, the PCR assays included conventional PCR assay to detect the 56kDa gene and real-time PCR assays to detect the 47kDa *htra* and *groEL* genes. To fulfil the PCR criteria for diagnosis, a consensus of two out of three PCR assays was required.

The inflammatory biomarker procalcitonin was measured by the ELISA-based VIDAS PCT kit with a detection range of 0.05-195ng/ml (BioMérieux, France), and CRP serum levels were measured with the NycoCard Reader II (Axis Shield, Norway), with a detection range of 5-150mg/L in serum [22, 23]. Testing was performed on admission samples and two independent operators, blinded to the microbiological diagnoses, performed the procalcitonin and CRP assays in duplicate. Control reagents were provided with each test kit and calibration performed as per manufacturers’ instructions. The following thresholds were evaluated for their usefulness in predicting bacterial causes of fever; for procalcitonin 0.25ng/mL and 0.5ng/mL, and for CRP 20mg/L and 40mg/L plasma levels upon admission, respectively [24–26].

### Attribution of final diagnosis

The diagnostic results were considered in relation to each other, and a final diagnosis was attributed to each case by the strength of evidence supporting each diagnosis, as previously described; (I) PCR/antigen/culture positivity > (II) dynamic serology (4-fold rise) > (III) single titer and/or unjustified serological cut-off titer [27].

Blood, urine, sputum and stool culture results from admission were collected from the hospital reporting system if performed. A final conservative diagnosis of culture-attributed infection (CAI) was made on the balance of clinical information, haematological and biochemical results, and results of our diagnostic panel.

### Statistical analysis

Proportions, percentages and averages (median and interquartile range [IQR] or mean and standard deviation [SD]) were calculated controlling for any missing data. Seasonality was assessed by calculating proportions of patients (and 95% confidence intervals) admitted during discrete time-periods and assessing for overlap as well as performing two-sample tests of proportions. Univariate and multivariate logistic regression analysis were performed to determine predictor variables independently associated with the outcomes (e.g. viral/bacterial/unknown aetiologies or specific diagnoses such as scrub typhus or dengue). Categorical data were analysed using Pearson’s Chi-squared test or Fisher’s exact test as appropriate where specified. Comparisons of receiver operating characteristic (ROC) curves evaluated the sensitivity, specificity and likelihood ratios for procalcitonin and CRP in differentiating between bacterial and viral aetiologies. Classification and regression trees were generated for scrub typhus and dengue using Salford Predictive Modeler Software Suite v8.2 (Salford Systems, San Diego, CA, USA). Other analyses were performed using STATA 14 software (College Station, Texas, USA).

## Results

### Demographic, clinical, diagnostic and laboratory findings

Our study cohort of 200 adult patients with AUF admitted to Chiangrai Prachanukroh hospital between August 2006 and October 2008 was predominantly male (114/194, 58.8%), had a median age of 41 (IQR 29–52), and most had a rural/agricultural occupation (64/136, 47.1%). 34/200 patients (17%) received antibiotic therapy prior to admission to the provincial hospital and the median number of days from onset of fever to admission was 4 (IQR 3–7).

77/200 patients (38.5%) had a bacterial aetiology for their fever, 24/200 (12%) a viral aetiology, and 97/200 (48.5%) had an unknown aetiology (the 2 remaining patients were diagnosed with invasive fungal infection, details below). Scrub typhus was the leading bacterial cause of AUF with 45/200 (22.5%), followed by leptospirosis with 15/200 (7.5%) and murine typhus 7/200 (3.5%), while dengue was the leading viral cause with 23/200 (11.5%) and there was a solitary JEV patient (0.5%).

A total of 12/200 (6%) cases had multiple positive tests (11 dual, 1 triple) that required scrutinizing with the criteria described above. Anti-JEV IgM positive cases were superseded by scrub typhus PCR positivity +/- dynamic serology in three cases and dengue NS1 antigen +/- dynamic serology in four cases. One case had weakly positive scrub typhus PCR for a single target (2 out of 3 targets required to fulfil the diagnostic criteria) with negative serology and was superseded by dengue NS1 antigen and IgM positivity. Two leptospirosis RDT positive cases were overruled by scrub typhus PCR-positivity in one case and dynamic murine typhus serology in the other. One case with dynamic rise in anti-dengue IgM but negative NS1 antigen was assigned a diagnosis of scrub typhus on the basis of PCR-positivity and dynamic serology. Finally, one case with positive leptospirosis RDT and anti-dengue IgM dynamic serology with negative NS1 antigen was diagnosed with scrub typhus on the basis of positive PCR assays.

142/200 (71%) patients had blood cultures performed of which, 126 were reported as no growth, 9 had microbiologically non-significant growth (mainly Gram positive organisms e.g. coagulase-negative staphylococci, aerobic spore bearers), and 7 had microbiologically significant growth (3.5%). Blood culture findings included 2 *Talaromyces marneffei*, 1 *Haemophilus influenzae*, 1 *Staphylococcus aureus*, 1 *Burkholderia pseudomallei*, 1 *Escherischia coli*, and 1 *Enterococcus faecium*. The patients with talaromycosis and *Haemophilus influenzae* bacteraemia tested positive for HIV antibodies using in-house RDTs. In addition, there were 2 significant urine cultures (heavy growth of *E*.*coli*), 2 significant sputum cultures (*Klebsiella pneumoniae* in patients with severe respiratory syndromes), and 1 significant stool culture (*Salmonella* spp.). In summary, there were 12 additional diagnoses in the culture-attributed infections group (CAI), 10 due to bacteria and 2 due to fungi.

Table 1 summarises the characteristics of patients in the viral, bacterial and unknown aetiology groups. Patients who were younger (OR 0.966, 95%CI 0.937–0.996, p = 0.026), had lower CRP (OR 0.967, 95% CI 0.953–0.981, p = 0.000), lower white blood count (OR 0.713, 95%CI 0.615–0.828, p = 0.000), lower neutrophil count (OR 0.694, 95%CI 0.586–0.822, p = 0.000) or higher haemoglobin (OR 1.259, 95%CI 1.023–1.549, p = 0.029) were significantly more likely to be diagnosed with a viral aetiology on univariate logistic regression analyses. Only low CRP (aOR 0.972, 95%CI 0.957–0.987, p = 0.000) and low white blood count (aOR 0.573, 95%CI 0.331–0.992, p = 0.047) remained as significant predictors for viral infection on multivariate logistic regression analysis. Significant predictor variables for bacterial infection on univariate analyses included the presence of an eschar (OR 11.74., 95%CI 3.849–35.807, p = 0.000) and a higher lymphocyte count (OR 1.366, 95%CI 1.027–1.816, p = 0.032) but only the eschar remained a significant predictor on multivariate analysis (aOR 11.590, 95%CI 3.754–35.784, p = 0.000). The finding of an eschar within the bacterial aetiology group was almost exclusively seen in patients diagnosed with scrub typhus (21/22, 95.5%), the exception being one patient with *Staphylococcus aureus* bacteraemia (1/22, 4.5%). Significant predictor variables for the unknown aetiology group are shown in Table 1 but are clinically less useful. Details of univariate and multivariate analyses of the predictor variables in Table 1 can be found in S1 Table. When comparing the viral and bacterial aetiology groups directly (excluding unknown group), eschar, CRP, Hb, WBC, neutrophil count and lymphocyte count were significant variables on univariate analyses. A lower CRP (aOR0.969 95%CI 0.951–0.987, p = 0.001) was an important predictor for viral infection while presence of an eschar (completely absent in the viral group) and a higher CRP (aOR1.032 95%CI 1.014–1.052, p = 0.001) remained as significant predictor variables for bacterial infection on multivariate analysis.

**Table 1:** Clinical, demographic and laboratory characteristics of patients by diagnostic group.

	*Viral aetiology (n = 24)*	*Bacterial aetiology (n = 77)*	*Unknown aetiology (n = 97)*
**Demographics and History**			
Male, n (%)	10/24 (41.7)	42/77 (54.5)	61/91 (67.0)^a^
Age, median (IQR)	32 (24–45)^a^	43 (28–50)	42 (30–54)
Rural occupation, n (%)	4/15 (26.7)	26/47 (55.3)	33/65 (50.8)
Pre-admission antibiotic, n (%)	2/8 (25.0)	14/42 (33.3)	18/54 (33.3)
Days with fever before admission, median (IQR)	5 (2.8–6)	5 (4–7)	4 (3–6)
Days of hospitalisation, median (IQR)	5.5 (5–7)	5 (4–7)	5 (4–7)
Clinical presentation*			
Fever, n (%)	23/24 (95.8)	75/77 (97.4)	92/97 (94.8)
Neurological findings n (%)	17/24 (70.8)	44/73 (60.3)	44/89 (49.4)
Respiratory findings n (%)	5/24 (20.8)	20/74 (27.0)	24/91 (26.4)
Gastrointestinal findings n (%)	16/24 (66.7)	37/73 (50.7)	47/90 (52.2)
Eschar, n (%)	0/24 (0.0)	22/74 (29.7)^a^^,^^b^	4/89 (4.5)^a^^,^^b^
Clinical severity, n (%)	6/24 (25)	14/74 (18.9)	20/91 (22.0)
Laboratory**			
CRP (mg/L), median (IQR)	12.5 (6.0–26.0)^a^^,^^b^	139.5 (67.5–150)	144 (56.0–150)^a^
Procalcitonin (ng/mL), median (IQR)	0.3 (0.1–1.3)	2.6 (0.9–7.3)	2.1 (0.4–24.0)^a^
Haemoglobin (g/dL), median (IQR)	13.2 (11.9–14.8)^a^	12 (10.6–13.0)	12.5 (11.0–14.0))
WBC (10^3^/mm^3^), median (IQR)	3 (2.1–5.6)^a^^,^^b^	10.6 (6.7–13.8)	9.8 (7.3–12.2)
Neutrophils (10^3^/mm^3^), median (IQR)	1.8 (1.0–3.4)^a^	7.7 (4.6–11.2)	7.3 (5.6–10.6)^a^
Lymphocytes (10^3^/mm^3^), median (IQR)	0.6 (0.4–1.2)	0.9 (0.5–1.8)^a^	0.8 (0.5–1.5)

^a^ Significant predictor variable on univariate logistic regression analysis

^b^ Significant predictor variable on multivariate logistic regression analysis

* Clinical presentation

Fever: tympanic temperature >37.5°C on or after admission

Neurological findings: at least one of meningism, headache, focal neurological deficits

Respiratory findings: at least one of respiratory rate >22/minute, lung crepitation, cough, dyspnoea

Gastrointestinal findings: at least one of abdominal pain, vomiting, nausea, jaundice, hepatomegaly, splenomegaly

Severity–at least one of these: intubation; respiratory rate >30/min; pulse >100/min; systolic blood pressure <90mmHg or >160mmHg, or diastolic blood pressure <60mmHg; haematemesis; haemoptysis, seizures, reduced GCS

**Laboratory reference range: CRP <10mg/L, PCT <0.1ng/mL, Hb 12-18g/dL, WBC 4.8–10.8x10^3^/mm^3^, N 2.6–7.0x10^3^/mm^3^, L 1.2–3.8x10^3^/mm^3^

For a breakdown of demographics, symptoms and signs, chest x-ray findings and detailed laboratory results for patients diagnosed with scrub typhus, dengue, leptospirosis and murine typhus, please refer to S2 Table. Significant predictors for scrub typhus on multivariate logistic regression analysis included the presence of an eschar (aOR 42.408, 95%CI 4.956–362.905, p = 0.001), a higher lymphocyte count (aOR 2.063, 95%CI 1.146–3.713, p = 0.016), and elevated aspartate aminotransferase (AST, aOR 1.014, 95%CI 1.004–1.023, p = 0.004) and alkaline phosphatase (ALP, aOR 1.004, 1.000–1.008, p = 0.036). For dengue, a lower CRP (aOR 0.956, 95%CI 0.927–0.986, p = 0.005) was the only consistently significant predictor variable on multivariate analysis. Elevated creatinine was significantly associated with leptospirosis on univariate analysis (OR 1.132, 95%CI 1.001–1.279, p = 0.048) but was not significant in multivariate analysis. Details of the analysis can be found in S3 Table. In addition, classification and regression trees (CART) were generated for scrub typhus (S1 Fig, panel A) and dengue (S1 Fig, panel B) which revealed a similar set of significant variables when compared with the multivariate logistic regression analyses above. The presence of an eschar, ALP>289IU/L and AST>88IU/L were used as decision nodes for scrub typhus while CRP≤37mg/L and WBC≤7.9x10^3^/mm^3^ were used for dengue virus.

**Fig 1:**
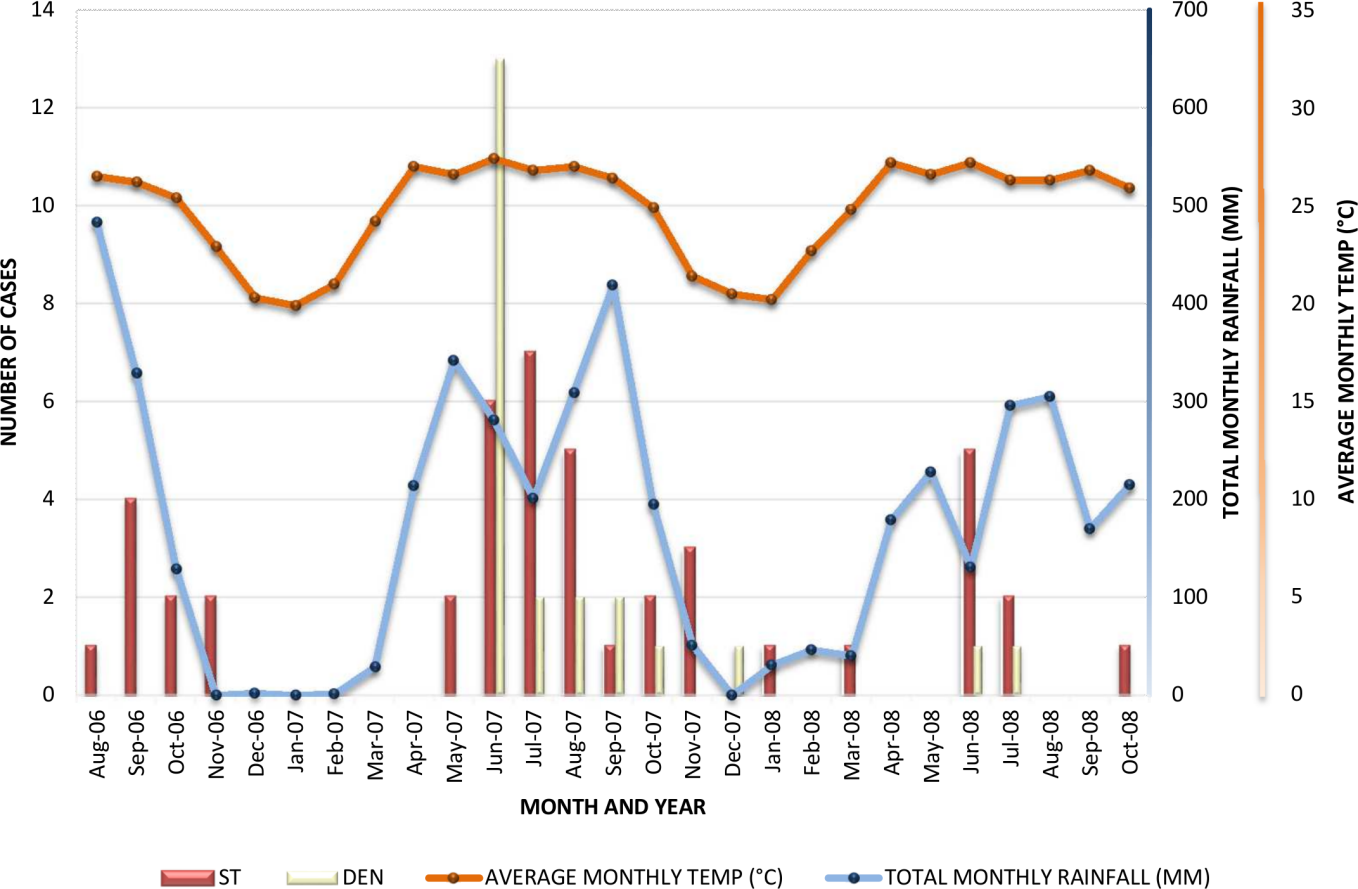
The temporal spread of scrub typhus and dengue cases and monthly meteorological data for Mueang district, Chiangrai province (Thai Meteorological Department, Chiangrai).

The majority of cases occurred during the months of June to November, coinciding with the rainy and early winter seasons. Proportions of patients (95% confidence intervals) admitted from June to November and from December to January were calculated for the study cohort, scrub typhus, dengue, leptospirosis, murine typhus, CAI and unknown groups: total 0.82 (0.77–0.88):0.18 (0.12–0.23) p<0.001, scrub typhus 0.91 (0.83–0.99):0.09 (0.01–0.17) p<0.001, dengue 0.96 (0.87–1.00):0.04 (0.00–0.13) p<0.001, leptospirosis 0.80 (0.60–1.00):0.20 (0.00–0.40) p = 0.001, murine typhus 0.57 (0.20–0.94):0.43 (0.06–0.80) p = 0.595, CAI 0.83 (0.62–1.00):0.17 (0.00–0.38) p = 0.04 and unknown 0.77 (0.68–0.86):0.23 (0.14–0.32) p<0.001. Apart from murine typhus, there were no overlaps of 95% confidence intervals. To illustrate further, scrub typhus and dengue cases were plotted against time along with average monthly temperatures and total monthly rainfall for the district (Fig 1).

A total of 9 deaths were recorded during this study. Three patients had a diagnosis of scrub typhus (3/45, 6.7%), two patients had bloodstream infections (*Talaromyces marneffei*–previously *Penicillium marneffei–*and *Haemophilus influenzae*, both HIV positive cases), while 4 patients had unknown aetiologies (4/97, 4.1%). The 9 patients consisted of 5 males and 4 females, with a median age of 44 (IQR 40–51), 7 worked in agriculture, none had received pre-admission antibiotics and all were treated with antibiotics upon admission with the exception of 1 patient who died soon after presentation. The median number of fever days prior to hospital admission was 4 (IQR 3–6) and the number of days admitted was 3 (IQR 2–4). The majority were febrile on or after admission (8/9, 88.9%) and had neurologic (7/8, 87.5%), respiratory (5/8, 62.5%), gastrointestinal (5/8, 62.5%) or severe disease (5/8, 62.5%). The median (IQR) CRP, PCT, WBC, neutrophil count values for this sub-group were 150 mg/L (149–150), 37.2 ng/mL (2.0–59.7), 10.2x10^3^/mm^3^ (8.3–14.8) and 9.3x10^3^/mm^3^ (6.9–12.2), respectively.

### Antimicrobial treatment regimens and prescriptions

169 out of 200 patients (84.5%) received antibiotics during the study (pre-admission and/or during admission). Of the 31 patients who did not receive any antibiotics, 6 had a viral infection (exclusively dengue), 7 had a bacterial infection (5 scrub typhus, 1 leptospirosis, and 1 bacteraemia) and 18 had an unknown aetiology. For monotherapy, ceftriaxone was the most commonly used antibiotic (131/169, 77.5%) followed by doxycycline (118/169, 69.8%) and chloramphenicol (26/169, 15.4%). Use of combination antibiotic therapy was common and particularly applied to patients during their in-patient stay 105/168 (62.5%) compared to those who received antibiotics prior to admission 9/34 (26.5%). Ceftriaxone and doxycycline was the most commonly used combination with 79/169 (46.7%) patients receiving this therapy.

Eighteen of twenty four patients (75%) with a viral diagnosis received antibiotics while patients with a bacterial diagnosis and those with an unknown aetiology received antibiotics in 93.3% (70/75) and 92.9% (79/85) of cases, respectively. Among patients with a diagnosis of scrub or murine typhus, 82.4% (42/51) received anti-rickettsial antibiotics (mainly doxycycline or chloramphenicol), which meant 17.6% of patients (9/51) received antibiotics ineffective against both diseases. In contrast, 93.3% of patients (14/15) with leptospirosis received appropriate treatment (ceftriaxone +/- doxycycline). An overview of antibiotic use is shown below in Table 2.

**Table 2:** Overview of prescribed antibiotics (before and during hospitalisation)*.

	3^rd^ generation Cephalosporin + anti-rickettsial antibiotic(s)**	Ceftriaxone only	Doxycycline only	Chloramphenicol only	Other anti-rickettsial treatment#	Other antibiotic (s)	None
**Scrub and murine typhus**	3.7	18.5	0	7.4	0	3.7	66.7
	52.9	7.8	19.6	7.8	2.0	2.0	7.8
**Leptospirosis**	0	12.5	0	0	0	0	87.5
	73.3	20.0	0	0	0	0	6.7
**Dengue and JEV**	0	25	0	0	0	0	75
	29.2	4.2	33.3	4.2	4.2	0	25
**CAI (bacterial)**	0	33.3	0	0	16.7	0	50
	66.7	33.3	0	0	0	0	0
**Unknown aetiology**	5.7	17.0	3.8	0	1.9	3.8	67.7
	50.0	16.7	14.3	2.4	3.6	6.0	7.1

* Percentages shown. Unshaded line = pre-hospital treatment, shaded line = inpatient treatment, CAI = culture-attributed infections

** Anti-rickettsial antibiotics = doxycycline, chloramphenicol, roxithromycin and ciprofloxacin (see discussion)

# Other anti-rickettsial treatment = a combination of the antibiotics above (e.g. doxycycline and chloramphenicol) or a combination of 1 anti-rickettsial antibiotic with other antibiotics (e.g. amoxicillin and roxithromycin)

Nevertheless, the strategy of combining a beta-lactam with doxycycline was often used, and 53%, 73% and 67% of patients with a rickettsiosis, leptospirosis and culture-attributed bacterial infection received an antimicrobial treatment regimen combining a third generation cephalosporin with a rickettsia-active drug, respectively. Ceftriaxone monotherapy was most commonly used for leptospirosis and bacterial causes, while doxycycline monotherapy was commonly used for the rickettsial/dengue subgroups.

### CRP and PCT biomarker results

CRP on admission was a significant predictor variable for the viral aetiology group (low CRP) when analysing the whole AUF cohort. When the unknown group was excluded, it remained an important predictor for the viral (low CRP) and bacterial (high CRP) groups. 92% and 86% of bacterial cases had CRP levels above the pre-defined cut-offs of >20mg/L and >40mg/L, respectively. For the viral aetiology group, 73% and 86% of cases had CRP levels below these cut-offs, respectively. The >20mg/L and >40mg/L CRP cut-offs correctly identified 87.2% and 86.2% of bacterial and viral cases, respectively. The CRP and PCT results are summarised in Table 3.

**Table 3:** CRP and PCT cut-off results.

Value	Bacterial aetiology n (%)	Viral aetiology n (%)	Sensitivity (95% CI)	Specificity (95% CI)	PPV (95% CI)	NPV (95% CI)	Correctly identified (%)
CRP >20 mg/L*	66/72 (91.7)	6/22 (27.3)	91.7 (82.7–96.9)	72.7 (49.8–89.3)	91.7 (82.7–96.9)	72.7 (49.8–59.3)	87.2%
CRP >40 mg/L*	62/72 (86.1)	3/22 (13.6)	86.1 (75.9–93.1)	86.4 (65.1–97.1)	95.4 (87.1–99.0)	65.5 (45.5–82.1)	86.2%
PCT >0.25 ng/mL*	65/72 (90.3)	13/22 (59.1)	90.3 (81–96.0)	40.9 (20.7–63.6)	83.3 (73.2–90.8)	56.3 (29.9–80.2)	78.7%
PCT >0.50 ng/mL*	58/72 (80.6)	8/22 (36.4)	80.6 (69.5–88.9)	63.6 (40.7–82.8)	87.9 (77.5–94.6)	50.0 (30.6–69.4)	76.6%

* Significant difference (p ≤0.001) between bacterial and viral groups at these cut-offs on direct comparison (Chi-squared test)

The optimal CRP plasma level cut-off to accurately distinguish between bacterial and viral causes for fever in this study was calculated to be >36mg/L [sensitivity 88.9% (95%CI 79.3–95.1) and specificity 86.4% (95%CI 65.1–97.1)], with 88.3% of cases correctly identified. If we compare the choice of CRP cut-offs according to available CRP assays of 20mg/L and 40mg/L, then using the 40mg/L cut-off would provide an improved balance between sensitivity and specificity, with a higher specificity than the lower cut-off of 20mg/L.

On excluding the unknown aetiology group, PCT was good at defining bacterial cases, but poor at selecting for viral aetiologies, which is reflected by the poor specificity values. The higher cut-off at 0.50ng/mL improved specificity from 40.9 to 63.6 when compared to 0.25ng/mL, and was accompanied with a moderate drop in sensitivity and a minor reduction in the proportion of correctly identified cases. If a higher cut-off of 0.7ng/mL for PCT was chosen, sensitivity will fall slightly while the specificity will increase, but with the same number of correctly identified cases [sensitivity 79.2% (68.0–87.8), specificity 68.2% (45.1–86.1), correctly identified cases 76.6%].

Receiver operating characteristic (ROC) curves were generated to visualise the performance of CRP (S2 Fig, panel A) and PCT (S2 Fig, panel B) in differentiating bacterial versus viral infections for specified cut-off values. The areas under the ROC curve were 0.91 (0.85–0.96, 95% CI) and 0.80 (0.72–0.88, 95% CI) for CRP and PCT, respectively.

## Discussion

In this study the cause of non-malarial AUF was determined in 51.5% of enrolled cases. Rickettsial illnesses (scrub typhus and murine typhus) continue to be leading causes of AUF in northern Thailand, and although awareness of these treatable illnesses is increasing at the hospital level–as reflected by the high proportion of cases correctly managed by local physicians—this is not the case at the community level where doxycycline is seldom used. It is notable that by deploying diagnostic tests for as few as five diseases and utilising conventional microbiological culture techniques in the local hospital microbiology laboratory, the causes of more than half of the AUF cases could be identified.

### Scrub typhus remains a leading cause of acute undifferentiated fever

Scrub typhus was the leading cause of AUF followed by dengue, leptospirosis, murine typhus, and bloodstream infections (22.5%, 11.5%, 7.5%, 3.5% and 3.5%, respectively) in this study. The incidence of both scrub typhus and dengue exhibited pronounced seasonality and were more common in the rainy season through to early winter (June to November). Similar to previous studies, the clinical finding of an eschar was strongly associated with the diagnosis of scrub typhus and represents a useful diagnostic clue [7, 28, 29]. However, eschars are not always present in scrub typhus patients, and their formation can be influenced by the degree of past exposure to various *Orientia tsutsugamushi* strains and the presence of strain-specific immunity [30]. Previous studies on paediatric scrub typhus in northern Thailand reported the presence of an eschar in approximately 70% of children [31, 32], while only 7% of children from Songkhla, southern Thailand and 7% of adults from Udon Thani, north-eastern Thailand with scrub typhus were reported to have an eschar [33, 34]. Whether this represents the spectrum of regular re-exposure to circulating *Orientia tsutsugamushi* strains in these regions remains to be determined in longitudinal studies.

Five patients presented with eschars but tested negative for scrub typhus. One patient had *Staphylococcus aureus* bacteraemia while the other four patients were in the unknown aetiology group. Additional testing of samples from one of these four patients revealed one 17kDa qPCR positive blood sample suggestive of *Rickettsia* spp. As such, alternative causes for febrile patients presenting with an eschar, such as spotted fever group rickettsial infections, should be considered. It is important to note that true eschars are completely painless–a central feature to distinguish them from eschar-like lesions including spider and (manipulated) insect bites which are typically itchy and/or painful [35].

In addition, we have shown that elevated hepatic enzymes (ALP and AST) were important predictors of scrub typhus in patients admitted with AUF on multivariate logistic regression and classification and regression tree (CART) analyses (ALP>289IU/L and AST>88IU/L). Raised hepatic enzymes have previously been described in scrub typhus observational studies in northern Thailand and India although not in cause of fever studies [36–38].

The overall mortality rate in our study cohort was 4.5% (total of 9 deaths) of which a third were attributable to scrub typhus. The scrub typhus mortality rate of 6.7% (3/45) was comparable to previous reports of untreated disease, as summarised in a recent systematic review, but was lower than the previously reported mortality from northern Thailand of 13.1% from 2004–2010, possibly reflecting better awareness and treatment decisions [36, 39].

### Antimicrobial treatment observations and implications

The majority of patients (84.5%) received empirical antibiotic treatment after admission to the provincial hospital, and 82.4% of patients subsequently diagnosed with scrub or murine typhus received an anti-rickettsial regimen. Doxycycline and chloramphenicol were the two main anti-rickettsial antibiotics used during the study and the majority of scrub typhus patients in our study recovered, despite previous reports of doxycycline and chloramphenicol resistant strains of *O*. *tsutsugamushi* in Chiangrai [40]. Of the 3 patients who died with scrub typhus, one did not receive any effective antimicrobial, one had delayed administration of chloramphenicol, and another received doxycycline and chloramphenicol from admission onwards. Azithromycin has been shown to be an effective alternative treatment in scrub typhus patients and appears also to be effective against resistant Chiangrai isolates of *O*. *tsutsugamushi* [41–43]. However, azithromycin was not used during this study due to the unavailability of more cost-effective generic formulations at the time. Nevertheless, the fact that 53% and 73% of patients with rickettsioses or leptospirosis, respectively, received a combination of a third generation cephalosporin plus a rickettsia-active antibiotic, and that in the remaining patients ceftriaxone was most commonly used for leptospirosis or bacterial causes, while doxycycline was commonly used for the rickettsial/dengue subgroups, demonstrates a high level of clinical experience and awareness among medical staff in this endemic area (Table 2).

Roxithromycin was used in 1 patient with scrub typhus in combination with doxycycline. There have been limited clinical studies into the effectiveness of roxithromycin in the treatment of scrub typhus [31, 44] and none reported for murine typhus. One case series from Chiangrai reported low efficacy of roxithromycin when compared to doxycycline or chloramphenicol in 20 children with scrub typhus [31]. *In vitro* susceptibility testing to roxithromycin has not been reported for *Orientia tsutsugamushi* while *Rickettsia typhi* appears susceptible [45]. Two patients with scrub typhus received ciprofloxacin, one as the sole anti-rickettsial antibiotic and the other in combination with doxycycline and chloramphenicol. Fluoroquinolones have been shown to be moderately effective in *in vitro* susceptibility tests and in limited clinical studies against murine typhus [45–47]. However, *Orientia tsutsugamushi* may be intrinsically resistant to fluoroquinolones which may explain the poor efficacy reported in clinical studies [48–51].

In contrast to antibiotic use in the hospital setting, only 34/200 (17%) of study patients received antibiotics prior to admission. Rickettsial infections make up 25% of patients presenting with AUF, and only 10 of 34 patients received pre-admission antibiotics with anti-rickettsial activity (5% of the study cohort)–of these only 5 patients received effective treatment for scrub typhus (2.5% of the study cohort). Supporting this observation prescription data from primary care units from the central Mueang district of Chiangrai province (2015) revealed low utilisation of anti-rickettsial antibiotics and doxycycline use was absent altogether. This highlights the need for improving the availability of specific antibiotics—particularly doxycycline—in rural endemic areas and for providing effective diagnostics to guide appropriate management of febrile patients, as inappropriate use of antibiotics has led to the development of antibiotic resistance, particularly impacting regions where access to effective antimicrobials is already limited [52].

### Differentiating between antibiotic treatable and non-treatable diseases–the role of biomarkers

The paucity of diagnostically useful clinical symptoms and signs in AUF cases should spur the development of affordable and effective rapid diagnostic tests (RDTs). Even at a provincial hospital in Thailand, it is unrealistic for costly and expertise-reliant tests such as IFAs, ELISAs and PCR assays to be performed routinely [53]. Previous studies have shown that robust and high-quality RDTs for common causes of AUF provide the best balance for diagnostic cost-effectiveness [15]. This though requires up-to-date and representative local epidemiological data–ideally based on fever surveillance studies. The provision of effective RDTs to diagnose scrub typhus, dengue and leptospirosis will cover 41% of cases of AUF presenting to the provincial hospital in Chiangrai. The use of algorithms incorporating both clinical findings with accurate RDTs +/- basic laboratory tests to guide early appropriate antibiotic management of patients presenting with AUF will likely improve this further.

In high income countries, biomarkers such as C-reactive protein (CRP) and procalcitonin, have been shown to be safe, cost-effective, and improve correct antibiotic use in the management of respiratory tract infections in primary care settings [17, 25, 54]. In Southeast Asia, it has been demonstrated that CRP can discriminate between bacterial and viral infections in acutely febrile patients and reduce antibiotic use in patients with non-severe respiratory tract infections in the community [18, 19]. Modelling the impact and cost-effectiveness of pathogen-specific RDTs and CRP tests using data from febrile outpatients in Laos revealed that tests for common prevalent bacterial infections (scrub typhus in that setting) and CRP levels were likely to be cost-effective for direct health benefits while tests for viral pathogens (e.g. dengue) were not [15].

This study demonstrated that low CRP and low WBC were significant predictors of a viral infection (mainly dengue, CRP≤37mg/L and WBC≤7.9x10^3^/mm^3^ on CART analysis). CRP was highly sensitive and very specific for defining bacterial infections (AUROC curve 0.9059), when directly comparing bacterial and viral groups, consistent with data from previous fever studies from Southeast Asia [18]. Currently, two CRP cut-offs are under investigation– 20mg/L and 40mg/L. The results in our study suggest that from a statistical point-of-view, choosing the higher cut-off improves specificity by almost 14%, thus reducing false positivity. However, this needs to be put into clinical context as the reduction of incorrectly treated viral cases from 6/22 (27.3%) to 3/22(13.6%) is offset by “missing” 4/72 (5.6%) of potentially severe bacterial cases–thus, reducing 3 cases with inappropriate antibiotic treatment comes at a cost of not treating 4 cases that would require antibiotics. If the test is employed at a community/primary care level, where monitoring facilities are limited, it could be argued that incorrectly treating an additional 3/22 (4.8%) febrile patients with a viral aetiology may be acceptable if an additional 4/72 (5.6%) patients with a potentially severe bacterial aetiology can be treated appropriately.

When comparing viral and bacterial groups, high procalcitonin was sensitive for the detection of bacterial infections but low levels were poor at selecting viral infections leading to low specificity. In Laos, elevated WBC counts have been shown to be significantly associated with fevers of bacterial aetiologies [6]. Previous fever studies from Southeast Asia have not specifically reported any association between neutrophilia and bacterial infections [3, 4, 6, 8, 55], although multiple reports have associated neutrophilia, lymphopaenia and elevated neutrophil-to-lymphocyte ratios with bacteraemic medical emergencies in high-income settings [56–58]. Our results suggest that simple laboratory tests such as full blood count and CRP could be beneficial in differentiating between bacterial and viral infections in acutely febrile patients at the hospital level, while a CRP-based POCT test (at USD 0.5–2.0 per test) is likely to be cost-effective in community settings in rural Southeast Asia. As Thailand expands its community health care system to fulfil one of five core priorities in partnership with the World Health Organization—this information is relevant to the development and commissioning of diagnostics in the community/district hospitals [59].

Although we were able to assign diagnoses to 51.5% of the febrile patient cohort, a large number of patients with unknown aetiologies demonstrated elevated laboratory markers described above and median CRP levels comparable to the diagnosed bacterial group–suggesting that a large proportion of potentially antibiotic treatable diseases go undiagnosed. The study has important limitations: i) due to budget constraints all cultures were performed in the local microbiology laboratory at the discretion of the treating physician; ii) there was an imbalance in the diagnostic investigations performed due to costs and limited access to high quality tests which may have led to bias (i.e. diagnostics for scrub typhus included PCR, culture and serology, while for leptospirosis only RDTs and culture were performed); and iii) the sample size is relatively small and external validity is limited although some conclusions can be drawn when the results are taken in context of previously published dataset from other fever studies from the region.

### Conclusion

In conclusion, this study has highlighted the importance of scrub typhus and dengue in the aetiology of AUF in Chiangrai province, northern Thailand. It has provided more evidence for including anti-rickettsial antibiotics into empirical hospital treatment guidelines and management strategies of AUF in the community. It contributes to the mounting evidence that good quality, accurate, pathogen-specific RDTs are urgently needed, which together with biomarker POCTs such as CRP, may aid healthcare workers in the correct use of antibiotics as part of the wider focus on antimicrobial stewardship. Finally, it emphasises the need for further prospective studies into the causes of AUF in the community along with evaluations of CRP POCTs in improving disease management algorithms, diagnostic accuracy, patient safety and reducing inappropriate antibiotic use in the tropics.

## Supporting information

S1 Fig Classification and regression trees (CART) for scrub typhus (A) and dengue (B).
(TIF)

S2 Fig ROC curves for plasma CRP levels (A) and plasma PCT levels (B) for differentiating between bacterial vs. viral infections.
(TIF)

S1 Table Results of univariate (A) and multivariate (B) logistic regression analyses for viral, bacterial and unknown aetiology groups.
(DOCX)

S2 Table Demographic, clinical, imaging and laboratory findings associated with scrub typhus, dengue, leptospirosis and murine typhus. (DOCX)

S3 Table Results of univariate (A) and multivariate (B) logistic regression analyses for scrub typhus, dengue, leptospirosis and murine typhus.
(DOCX)

S4 Table STROBE Statement and checklist for this study. (DOCX)
