# Supplementary figures and images for: Causes of acute undifferentiated fever and the utility of biomarkers in Chiangrai, northern Thailand
Source: PLoS Negl Trop Dis. 2018 May 31;12(5):e0006477. doi: 10.1371/journal.pntd.0006477 (PMC5978881; doi:10.1371/journal.pntd.0006477)

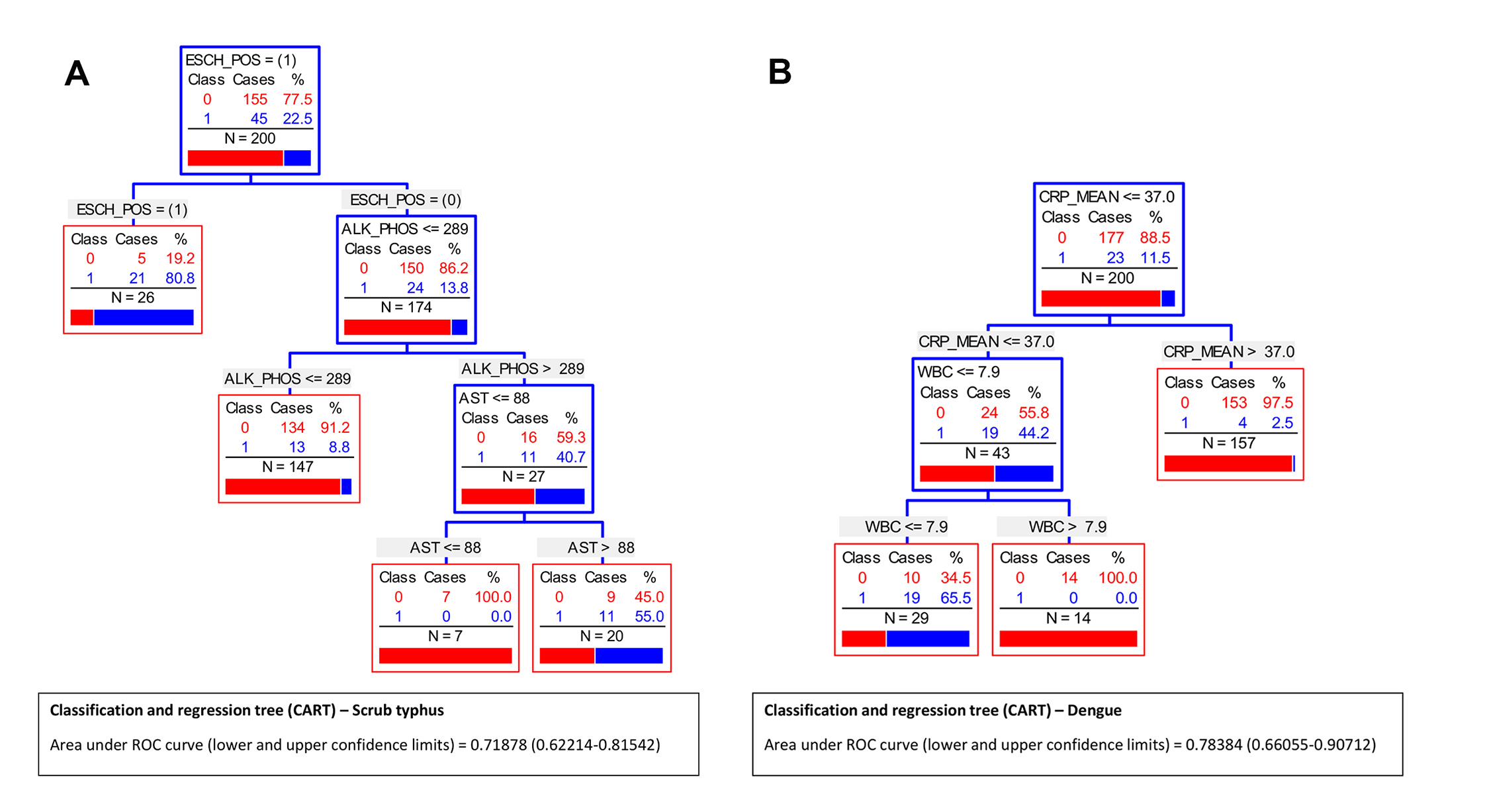

Supplement: S1 Fig — Classification and regression trees (CART) for scrub typhus (A) and dengue (B). (TIF) [file pntd.0006477.s001.tif]

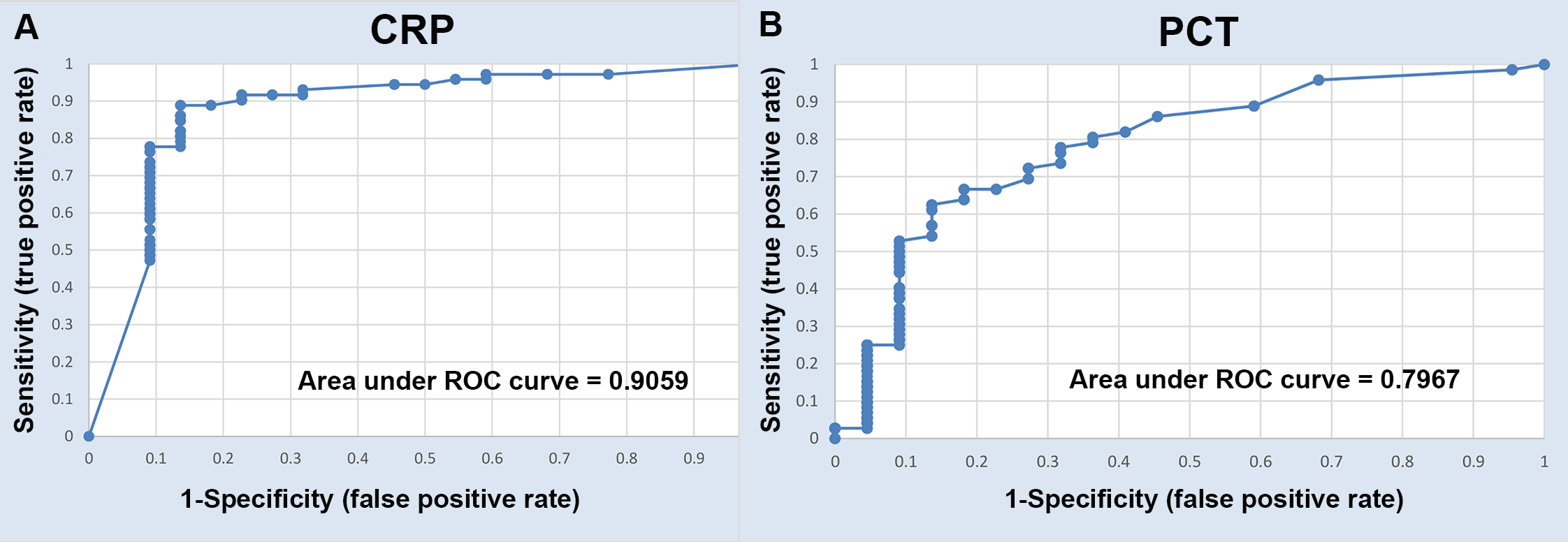

Supplement: S2 Fig — ROC curves for plasma CRP levels (A) and plasma PCT levels (B) for differentiating between bacterial vs. viral infections. (TIF) [file pntd.0006477.s002.tif]
