# Supplementary material for: Causes of acute undifferentiated fever and the utility of biomarkers in Chiangrai, northern Thailand
Source: PLoS Negl Trop Dis. 2018 May 31;12(5):e0006477. doi: 10.1371/journal.pntd.0006477 (PMC5978881; doi:10.1371/journal.pntd.0006477)
Supplement: S1 Table — Results of univariate (A) and multivariate (B) logistic regression analyses for viral, bacterial and unknown aetiology groups. (DOCX) [file pntd.0006477.s003.docx]

|  | ***Univariate logistic regression analyses*** | | | | | | | | |
| --- | --- | --- | --- | --- | --- | --- | --- | --- | --- |
| **S1 Table (A)** | ***Viral aetiology*** | | | ***Bacterial aetiology*** | | | ***Unknown aetiology*** | | |
|  | **OR** | **95% CI OR** | **P value** | **OR** | **95% CI OR** | **P value** | **OR** | **95% CI OR** | **P value** |
| ***Demographics and History*** | | | | | | | | | |
| Sex (M=1, F=2) | 2.206 | 0.926-5.256 | 0.074 | 1.333 | 0.744-2.389 | 0.334 | 0.521 | 0.291-0.934 | 0.029* |
| Age | 0.966 | 0.937-0.996 | 0.026* | 0.996 | 0.978-1.015 | 0.706 | 1.016 | 0.998-1.035 | 0.084 |
| Rural occupation | 3.113 | 0.935-10.362 | 0.064 | 0.714 | 0.347-1.469 | 0.360 | 0.939 | 0.470-1.879 | 0.860 |
| Pre-admission antibiotic | 0.688 | 0.131-3.598 | 0.657 | 1.100 | 0.479-2.526 | 0.822 | 1.125 | 0.497-2.546 | 0.777 |
| Fever days before admission | 0.892 | 0.736-1.082 | 0.246 | 1.114 | 0.998-1.242 | 0.053 | 0.943 | 0.848-1.050 | 0.286 |
| Days of hospitalisation | 1.023 | 0.911-1.150 | 0.698 | 0.982 | 0.893-1.081 | 0.717 | 1.012 | 0.924-1.108 | 0.800 |
| ***Clinical presentation*** | | | | | | | | | |
| Fever | 0.953 | 0.112-8.098 | 0.965 | 1.923 | 0.378-9.779 | 0.431 | 0.552 | 0.128-2.375 | 0.425 |
| Neurological findings | 0.542 | 0.222-1.322 | 0.178 | 1.686 | 0.929-3.059 | 0.086 | 0.797 | 0.448-1.416 | 0.439 |
| Respiratory findings | 0.736 | 0.259-2.089 | 0.564 | 1.124 | 0.579-2.181 | 0.730 | 1.075 | 0.561-2.058 | 0.828 |
| Gastrointestinal findings | 1.837 | 0.745-4.528 | 0.186 | 0.806 | 0.448-1.451 | 0.473 | 0.874 | 0.493-1.551 | 0.646 |
| Eschar | - | - | - | 11.740 | 3.849-35.807 | 0.000* | 0.167 | 0.055-0.506 | 0.002* |
| Clinical severity | 1.257 | 0.464-3.404 | 0.653 | 0.778 | 0.377-1.603 | 0.496 | 1.060 | 0.531-2.115 | 0.869 |
| ***Laboratory*** | | | | | | | | | |
| CRP | 0.967 | 0.953-0.981 | 0.000* | 1.005 | 1.000-1.011 | 0.060 | 1.005 | 1.000-1.011 | 0.042* |
| PCT | 0.945 | 0.887-1.007 | 0.081 | 0.992 | 0.979-1.005 | 0.213 | 1.014 | 1.001-1.028 | 0.033* |
| Hb | 1.259 | 1.023-1.549 | 0.029* | 0.881 | 0.769-1.009 | 0.066 | 1.035 | 0.910-1.177 | 0.600 |
| WBC | 0.713 | 0.615-0.828 | 0.000* | 1.040 | 0.986-1.096 | 0.153 | 1.054 | 0.999-1.113 | 0.056 |
| Neutrophil count | 0.694 | 0.586-0.822 | 0.000* | 1.032 | 0.974-1.094 | 0.286 | 1.067 | 1.004-1.134 | 0.037* |
| Lymphocyte count | 0.537 | 0.277-1.041 | 0.066 | 1.366 | 1.027-1.816 | 0.032* | 0.912 | 0.694-1.199 | 0.510 |

**Significant predictor variable on univariate logistic regression analysis*

*NB – Unless specified, for binary categorical variables, 0=no or absent, 1=yes or present*

| **S2 Table (B)** | ***Significant predictor variables associated with each aetiology group on multivariate logistic regression analysis*** | | |
| --- | --- | --- | --- |
|  | **aOR** | **95% CI OR** | **P value** |
| *Viral aetiology*   - WBC - CRP | 0.573  0.972 | 0.331-0.992  0.957-0.987 | 0.047  0.000 |
| *Bacterial aetiology*   - Eschar | 11.590 | 3.754-35.784 | 0.000 |
| *Unknown aetiology*   - Eschar | 0.148 | 0.041-0.535 | 0.004 |

*NB – Unless specified, for binary categorical variables, 0=no or absent, 1=yes or present*
