# Supplementary material for: Causes of acute undifferentiated fever and the utility of biomarkers in Chiangrai, northern Thailand
Source: PLoS Negl Trop Dis. 2018 May 31;12(5):e0006477. doi: 10.1371/journal.pntd.0006477 (PMC5978881; doi:10.1371/journal.pntd.0006477)
Supplement: S2 Table — (DOCX) [file pntd.0006477.s004.docx]

| **S2 Table** | ***Diagnosis*** | | | |
| --- | --- | --- | --- | --- |
|  | ***Scrub typhus*** | ***Dengue*** | ***Leptospirosis*** | ***Murine typhus*** |
| No. of patients | 45 | 23 | 15 | 7 |
| ***Demographics*** |  |  |  |  |
| Male, n (%) | 24/45 (53.3) | 9/23 (39.1)^a^ | 11/15 (73.3) | 5/7 (71.4) |
| Age, median (IQR) | 41 (28-47) | 29 (24-45)^a^ | 33 (22-50) | 49 (41-54) |
| Rural occupation, n (%) | 16/27 (59.3) | 4/14 (28.6) | 6/11 (54.5) | 1/2 (50.0) |
| Pre-admission antibiotic, n (%) | 7/23 (30.4) | 2/8 (25.0) | 2/9 (22.2) | 2/4 (50.0) |
| Days with fever before admission, median (IQR) | 6 (4-8)^a^ | 5 (3-6) | 5 (4-7) | 5 (3-7) |
| Days of hospitalization, median (IQR) | 5 (4-7) | 5 (5-7) | 5 (3.5-7) | 5 (4-7) |
| ***Symptoms and signs*** |  |  |  |  |
| Eschar | 21 (46.7)^a,b^ | 0 (0) | 0 (0) | 0 (0) |
| Rash | 1 (2.2) | 3 (13.0) | 0 (0) | 0 (0) |
| Cough/dyspnoea | 15 (33.3)^a^ | 3 (13.0) | 2 (13.3) | 0 (0) |
| Lung crepitation | 5 (11.1) | 1 (4.3) | 0 (0) | 0 (0) |
| Epistaxis | 0 (0) | 0 (0) | 1 (6.7) | 0 (0) |
| Haemoptysis | 0 (0) | 0 (0) | 0 (0) | 0 (0) |
| Nausea/vomiting | 6 (13.3) | 9 (39.1)^a,b^ | 3 (20.0) | 1 (14.3) |
| Abdominal pain | 12 (26.7) | 8 (34.8) | 5 (33.3) | 3 (42.9) |
| Jaundice | 8 (17.8) | 2 (8.7) | 3 (20.0) | 1 (14.3) |
| Hepatomegaly | 14 (31.1)^a^ | 7 (30.4) | 1 (6.7) | 0 (0) |
| Splenomegaly | 2 (4.4) | 0 (0) | 0 (0) | 0 (0) |
| Gum bleeding | 0 (0) | 1 (4.3) | 0 (0) | 0 (0) |
| Haematemesis | 0 (0) | 1 (4.3) | 0 (0) | 0 (0) |
| Headache | 26 (57.8) | 15 (65.2) | 7 (46.7) | 6 (85.7) |
| Conjunctivitis | 11 (24.4) | 3 (13.0) | 5 (33.3) | 1 (14.3) |
| Conjunctival haemorrhage | 1 (2.2) | 1 (4.3) | 1 (6.7) | 0 (0) |
| Tinnitus | 2 (4.4) | 0 (0) | 0 (0) | 0 (0) |
| Deafness | 0 (0) | 0 (0) | 0 (0) | 0 (0) |
| Neck stiffness | 2 (4.4) | 2 (8.7) | 1 (6.7) | 0 (0) |
| Myalgia | 15 (33.3) | 4 (17.4)^a^ | 6 (40.0) | 3 (42.9) |
| Lymphadenopathy | 1 (2.2) | 0 (0) | 0 (0) | 0 (0) |
| **Chest X-ray findings** |  |  |  |  |
| Performed, n (%) | 32 (71.1) | 14 (60.9) | 11 (73.3) | 4 (57.1) |
| - Normal or incidental findings, n (%) | 24 (75.0) | 13 (92.9) | 9 (81.8) | 4 (100.0) |
| - Pulmonary infiltrates | 7 (21.9) | 0 (0) | 2 (18.2) | 0 (0) |
| - Pulmonary oedema | 1 (3.1) | 0 (0) | 0 (0) | 0 (0) |
| - Pleural effusion | 0 (0) | 1 (7.1) | 0 (0) | 0 (0) |
| **Laboratory findings** |  |  |  |  |
| Haemoglobin (g/dL), median (IQR) | 11.9 (9.9-13.0) | 13.3 (12.3-14.9)^a^ | 12.2 (11.0-13.8) | 13.3 (12.9-15.0) |
| WBC (10^3^/mm^3^), median (IQR) | 9.5 (6.4-12.3) | 2.9 (1.9-5.1)^a^ | 10.9 (6.6-16.9) | 9.9 (6.9-12.8) |
| Neutrophils (10^3^/mm^3^), median (IQR) | 6.4 (4.3-10.5) | 1.7 (0.8-3.3)^a^ | 8.6 (4.9-14.9) | 8.1 (3.9-10.8) |
| Lymphocytes (10^3^/mm^3^), median (IQR) | 1.2 (0.5-2.3)^a,b^ | 0.7 (0.4-1.3)^a^ | 0.9 (0.4-1.1) | 0.8 (0.6-2.0) |
| Platelets (10^3^/mm^3^), median (IQR) | 94 (61-170) | 81 (25-101)^a^ | 96 (20-169) | 72 (29-203) |
| BUN (mg/dL), median (IQR) | 15.0 (12.0-28.0) | 12.0 (7.3-14.8)^a^ | 30.0 (17.0-82.5) | 21.0 (10.0-24.0) |
| Creatinine (mg/dL), median (IQR) | 1.0 (0.9-1.6) | 1.1 (0.8-2.3) | 3.6 (1.2-6.3)^a^ | 1.3 (1.3-2.0) |
| Bilirubin total (mg/dL), median (IQR) | 1.8 (0.9-3.1) | 0.6 (0.5-1.8) | 1.2 (0.6-7.2) | 0.8 (0.6-2.3) |
| Bilirubin direct (mg/dL), median (IQR) | 1.0 (0.3-1.8) | 0.2 (0.1-0.8) | 0.6 (0.1-5.1) | 0.3 (0.1-1.5) |
| AST (IU/L), median (IQR) | 148 (96-304)^a,b^ | 94 (57-240) | 43 (27-68) | 115 (63-303) |
| ALT (IU/L), median (IQR) | 101 (54-189)^a^ | 58 (29-95) | 41 (28-62) | 69 (44-333) |
| ALP (IU/L), median (IQR) | 271 (160-422)^a,b^ | 108 (78-134)^a^ | 127 (111-187) | 132 (109-249) |
| Albumin (g/dL), median (IQR) | 2.9 (2.5-3.2) | 3.6 (3.1-3.8) | 3.3 (2.7-3.7) | 3.4 (1.8-4.2) |
| CRP (mg/L), median (IQR) | 130.5 (67.5-150) | 12.0 (5.5-30.3)^a,b^ | 150 (60.3-150) | 103.0 (60.0-150) |
| PCT (ng/mL), median (IQR) | 2.5 (1.0-6.6) | 0.3 (0.1-1.4) | 4.7 (2.7-25.2) | 1.2 (0.2-3.7) |

- *^a^ Significant predictor variable on univariate logistic regression analysis*
- *^b^ Significant predictor variable on multivariate logistic regression analysis*
- Rural occupation included farmers, gardeners, agricultural/plantation workers, fish and animal farm workers
- Incidental findings on CXR included hyperinflation, hiatus hernia, cardiomegaly without accompanying pulmonary signs, ground glass/fibrotic changes
- WBC, white blood cell count; BUN, blood urea nitrogen; AST, aspartate aminotransferase; ALT, alanine aminotransferase; ALP, alkaline phosphatase; CRP, C-reactive protein; PCT, procalcitonin
- Laboratory reference range:
  - Hb – 12-18g/dL
  - WBC – 4.8-10.8x10^3^/mm^3^
  - Neutrophils - 2.6-7.0x10^3^/mm^3^
  - Lymphocytes - 1.2-3.8x10^3^/mm^3^
  - Platelets – 130-400x10^3^/mm^3^
  - BUN – 8-20mg/dL
  - Creatinine – 0.51-0.95mg/dL
  - Bilirubin total – 0.3-1.2mg/dL
  - Bilirubin direct – 0-0.2mg/dL
  - AST – 0-35IU/L
  - ALT – 0-35IU/L
  - ALP – 30-120IU/L
  - Albumin – 3.5-5.2g/dL
  - CRP – <10mg/L
  - PCT – <0.1ng/mL
