# Supplementary material for: Causes of acute undifferentiated fever and the utility of biomarkers in Chiangrai, northern Thailand
Source: PLoS Negl Trop Dis. 2018 May 31;12(5):e0006477. doi: 10.1371/journal.pntd.0006477 (PMC5978881; doi:10.1371/journal.pntd.0006477)
Supplement: S3 Table — Results of univariate (A) and multivariate (B) logistic regression analyses for scrub typhus, dengue, leptospirosis and murine typhus. (DOCX) [file pntd.0006477.s005.docx]

| **S3 Table (A)** | ***Univariate logistic regression analyses*** | | | | | | | | | | | |
| --- | --- | --- | --- | --- | --- | --- | --- | --- | --- | --- | --- | --- |
|  | ***Scrub typhus*** | | | ***Dengue*** | | | ***Leptospirosis*** | | | ***Murine typhus*** | | |
|  | **OR** | **95% CI OR** | **P value** | **OR** | **95% CI OR** | **P value** | **OR** | **95% CI OR** | **P value** | **OR** | **95% CI OR** | **P value** |
| ***Demographics*** | | | | | | | | | | | | |
| Sex (M=1, F=2) | 1.335 | 0.682-2.612 | 0.399 | 2.475 | 1.014-6.040 | 0.047* | 0.493 | 0.151-1.607 | 0.241 | 0.559 | 0.106-2.956 | 0.494 |
| Age | 0.983 | 0.961-1.006 | 0.142 | 0.965 | 0.936-0.996 | 0.027* | 0.976 | 0.941-1.012 | 0.181 | 1.040 | 0.993-1.090 | 0.099 |
| Rural occupation | 0.623 | 0.263-1.473 | 0.281 | 2.778 | 0.823-9.375 | 0.100 | 0.819 | 0.237-2.833 | 0.753 | 1.000 | 0.061-16.342 | 1.000 |
| Pre-admission antibiotic | 0.907 | 0.334-2.466 | 0.849 | 0.688 | 0.131-3.598 | 0.657 | 0.580 | 0.114-2.955 | 0.512 | 2.188 | 0.295-16.230 | 0.444 |
| Fever days before admission | 1.164 | 1.033-1.312 | 0.013* | 0.905 | 0.746-1.098 | 0.310 | 1.066 | 0.905-1.255 | 0.443 | 0.968 | 0.746-1.256 | 0.809 |
| Days of hospitalisation | 1.036 | 0.935-1.148 | 0.497 | 1.017 | 0.901-1.147 | 0.790 | 0.960 | 0.793-1.162 | 0.675 | 0.952 | 0.724-1.253 | 0.727 |
| ***Symptoms and signs*** | | | | | | | | | | | | |
| Eschar | 26.918 | 9.198-78.777 | 0.000* | - | - | - | - | - | - | - | - | - |
| Rash | 0.299 | 0.038-2.388 | 0.255 | 2.600 | 0.649-10.408 | 0.177 | - | - | - | - | - | - |
| Cough/dyspnoea | 2.469 | 1.158-5.263 | 0.019* | 0.488 | 0.138-1.731 | 0.267 | 0.515 | 0.112-2.380 | 0.396 |  |  |  |
| Lung crepitation | 3.171 | 0.916-10.984 | 0.069 | 0.686 | 0.084-5.626 | 0.726 | - | - | - | - | - | - |
| Epistaxis | - | - | - | - | - | - | 5.929 | 0.506-69.507 | 0.156 | - | - | - |
| Haemoptysis | - | - | - | - | - | - | - | - | - | - | - | - |
| Nausea/vomiting | 0.556 | 0.215-1.433 | 0.224 | 2.829 | 1.120-7.144 | 0.028* | 0.931 | 0.249-3.475 | 0.915 | 0.614 | 0.072-5.256 | 0.656 |
| Abdominal pain | 1.093 | 0.507-2.353 | 0.821 | 1.451 | 0.575-3.665 | 0.431 | 1.486 | 0.473-4.666 | 0.498 | 2.688 | 0.524-13.775 | 0.236 |
| Jaundice | 1.095 | 0.455-2.637 | 0.840 | 0.399 | 0.089-1.789 | 0.230 | 1.153 | 0.307-4.332 | 0.833 | 0.753 | 0.088-6.462 | 0.796 |
| Hepatomegaly | 3.182 | 1.395-7.256 | 0.006* | 2.174 | 0.791-5.973 | 0.132 | 0.339 | 0.042-2.749 | 0.311 | - | - | - |
| Splenomegaly | 7.077 | 0.625-80.115 | 0.114 | - | - | - | - | - | - | - | - | - |
| Gum bleeding | - | - | - | 3.810 | 0.331-43.846 | 0.283 | - | - | - | - | - | - |
| Haematemesis | - | - | - | 7.667 | 0.462-127.198 | 0.155 | - | - | - | - | - | - |
| Headache | 1.347 | 0.664-2.731 | 0.409 | 2.098 | 0.774-5.683 | 0.145 | 0.654 | 0.227-1.887 | 0.432 | 4.875 | 0.575-41.351 | 0.146 |
| Conjunctivitis | 1.651 | 0.736-3.704 | 0.224 | 0.582 | 0.163-2.075 | 0.404 | 2.219 | 0.710-6.937 | 0.171 | 0.676 | 0.079-5.792 | 0.721 |
| Conjunctival haemorrhage | 0.690 | 0.078-6.078 | 0.738 | 1.495 | 0.167-13.425 | 0.719 | 2.343 | 0.256-21.470 | 0.451 | - | - | - |
| Tinnitus | 7.282 | 0.643-82.424 | 0.109 | - | - | - | - | - | - | - | - | - |
| Deafness | - | - | - | - | - | - | - | - | - | - | - | - |
| Neck stiffness | 1.198 | 0.232-6.185 | 0.829 | 2.365 | 0.448-12.491 | 0.311 | 1.881 | 0.213-16.572 | 0.569 | - | - | - |
| Myalgia | 0.738 | 0.361-1.505 | 0.403 | 0.278 | 0.090-0.857 | 0.026* | 0.943 | 0.321-2.769 | 0.915 | 1.068 | 0.232-4.918 | 0.932 |
| Lymphadenopathy | 0.566 | 0.067-4.812 | 0.602 | - | - | - | - | - | - | - | - | - |
| ***CXR*** | | | | | | | | | | | | |
| Pulmonary infiltrates | 2.062 | 0.723-5.882 | 0.176 | - | - | - | 1.347 | 0.266-6.814 | 0.719 | - | - | - |
| ***Laboratory findings*** | | | | | | | | | | | | |
| Hb | 0.873 | 0.746-1.020 | 0.087 | 1.286 | 1.039-1.591 | 0.021* | 1.002 | 0.786-1.278 | 0.985 | 1.354 | 0.943-1.944 | 0.101 |
| WBC | 1.001 | 0.940-1.065 | 0.987 | 0.688 | 0.586-0.807 | 0.000* | 1.042 | 0.957-1.134 | 0.339 | 1.017 | 0.894-1.157 | 0.797 |
| Neutrophils | 0.984 | 0.917-1.056 | 0.650 | 0.673 | 0.562-0.807 | 0.000* | 1.057 | 0.967-1.155 | 0.223 | 1.049 | 0.917-1.199 | 0.488 |
| Lymphocytes | 1.538 | 1.134-2.085 | 0.006* | 0.470 | 0.225-0.981 | 0.044* | 0.552 | 0.237-1.286 | 0.169 | 1.087 | 0.569-2.075 | 0.801 |
| Platelets | 1.000 | 1.000-1.000 | 0.501 | 1.000 | 1.000-1.000 | 0.027* | 1.000 | 1.000-1.000 | 0.339 | 1.000 | 1.000-1.000 | 0.459 |
| BUN | 0.996 | 0.984-1.008 | 0.501 | 0.968 | 0.937-1.000 | 0.049* | 1.013 | 1.000-1.027 | 0.055 | 0.981 | 0.943-1.021 | 0.349 |
| Creatinine | 0.920 | 0.799-1.059 | 0.245 | 0.964 | 0.817-1.138 | 0.666 | 1.132 | 1.001-1.279 | 0.048* | 0.885 | 0.612-1.279 | 0.515 |
| Bilirubin total | 1.016 | 0.980-1.054 | 0.394 | 0.975 | 0.890-1.069 | 0.593 | 1.007 | 0.952-1.065 | 0.808 | 0.755 | 0.413-1.382 | 0.363 |
| Bilirubin direct | 1.021 | 0.925-1.126 | 0.684 | 0.925 | 0.746-1.146 | 0.476 | 1.063 | 0.943-1.200 | 0.317 | 0.750 | 0.353-1.597 | 0.456 |
| AST | 1.003 | 1.000-1.005 | 0.017* | 1.002 | 0.999-1.004 | 0.144 | 0.988 | 0.975-1.001 | 0.063 | 1.001 | 0.997-1.005 | 0.655 |
| ALT | 1.004 | 1.001-1.007 | 0.007* | 1.001 | 0.997-1.004 | 0.795 | 0.989 | 0.973-1.005 | 0.162 | 1.003 | 0.999-1.008 | 0.177 |
| Alk phos | 1.005 | 1.002-1.008 | 0.000* | 0.991 | 0.983-0.999 | 0.020* | 0.997 | 0.992-1.003 | 0.322 | 0.999 | 0.993-1.005 | 0.798 |
| Albumin | 1.024 | 0.979-1.072 | 0.292 | 0.989 | 0.902-1.084 | 0.814 | 0.973 | 0.787-1.205 | 0.805 | 0.714 | 0.210-2.431 | 0.590 |
| CRP | 1.003 | 0.997-1.010 | 0.313 | 0.968 | 0.954-0.982 | 0.000* | 1.006 | 0.995-1.017 | 0.300 | 1.001 | 0.988-1.015 | 0.869 |
| PCT | 0.983 | 0.963-1.004 | 0.107 | 0.948 | 0.891-1.009 | 0.091 | 1.012 | 0.996-1.027 | 0.133 | 0.980 | 0.928-1.035 | 0.475 |

**Significant predictor variable on univariate logistic regression analysis*

*NB – Unless specified, for binary categorical variables, 0=no or absent, 1=yes or present*

| **S3 Table (B)** | ***Significant predictor variables associated with each diagnosis on multivariate logistic regression analysis*** | | |
| --- | --- | --- | --- |
|  | **aOR** | **95% CI OR** | **P value** |
| *Scrub typhus*   - Eschar - Lymphocytes - AST - Alk phos | 42.408  2.063  1.014  1.004 | 4.956-362.905  1.146-3.713  1.004-1.023  1.000-1.008 | 0.001  0.016  0.004  0.036 |
| *Dengue*   - CRP | 0.956 | 0.927-0.986 | 0.005 |

*NB – no variables were significantly associated with leptospirosis or murine typhus on multivariate logistic regression analysis in this study*

*– Unless specified, for binary categorical variables, 0=no or absent, 1=yes or present*
